# Complementary alternative medicine use among patients with dengue fever in the hospital setting: a cross-sectional study in Malaysia

**DOI:** 10.1186/s12906-016-1017-0

**Published:** 2016-01-29

**Authors:** SiewMooi Ching, Vasudevan Ramachandran, Lai Teck Gew, Sazlyna Mohd Sazlly Lim, Wan Aliaa Wan Sulaiman, Yoke Loong Foo, Zainul Amiruddin Zakaria, Nurul Huda Samsudin, Paul Chih Ming Chih Lau, Sajesh K. Veettil, Fankee Hoo

**Affiliations:** 1 Department of Family Medicine, Faculty of Medicine and Health Sciences, Universiti Putra Malaysia, Serdang, Malaysia; 2 Malaysian Research Institute on Ageing, Universiti Putra Malaysia, 43400 Serdang, Malaysia; 3 Department of Medicine, Hospital Kuala Lumpur, Kuala Lumpur, Malaysia; 4 Department of Medicine, Faculty of Medicine and Health Sciences, University Putra Malaysia, Serdang, 43400 Selangor Malaysia; 5 Department of Biomedical Science, Faculty of Medicine and Health Sciences, Universiti Putra Malaysia, Serdang, Selangor 43400 Malaysia; 6 Department of Pharmacy Practice, International Medical University, Kuala Lumpur, 57000 Malaysia

**Keywords:** Complementary alternative medicine, Dengue fever, Prevalence, Hospital, Malaysia

## Abstract

**Background:**

In Malaysia, the number of reported cases of dengue fever demonstrates an increasing trend. Since dengue fever has no vaccine or antiviral treatment available, it has become a burden. Complementary and alternative medicine (CAM) has become one of the good alternatives to treat the patients with dengue fever. There is limited study on the use of CAM among patients with dengue fever, particularly in hospital settings. This study aims to determine the prevalence, types, reasons, expenditure, and resource of information on CAM use among patients with dengue fever.

**Methods:**

This is a descriptive, cross-sectional study of 306 patients with dengue fever, which was carried out at the dengue clinic of three hospitals. Data were analysed using IBM SPSS Statistics version 21.0 and logistic regression analysis was used to determine the factors associated with CAM use.

**Results:**

The prevalence of CAM use was 85.3 % among patients with dengue fever. The most popular CAMs were isotonic drinks (85.8 %), crab soup (46.7 %) and papaya leaf extract (22.2 %). The most common reason for CAM use was a good impression of CAM from other CAM users (33.3 %). The main resource of information on CAM use among patients with dengue fever was family (54.8 %). In multiple logistic regression analysis, dengue fever patients with a tertiary level are more likely to use CAM 5.8 (95 % confidence interval (CI 1.62–20.45) and 3.8 (95 % CI 1.12–12.93) times than secondary level and primary and below respectively.

**Conclusion:**

CAM was commonly used by patients with dengue fever. The predictor of CAM use was a higher level of education.

**Electronic supplementary material:**

The online version of this article (doi:10.1186/s12906-016-1017-0) contains supplementary material, which is available to authorized users.

## Background

Complementary and alternative medicine (CAM) is a group of various medical and healthcare systems, products, practices, approaches, knowledge and beliefs, which are not universally considered as a part of conventional Western medicine for maintaining well-being [1, 2]. The National Center for Complementary and Integrative Health classified CAM into five subdivisions: biological-based therapies, alternative medical systems, energy therapies, manipulative and body-based systems, and mind-body interventions [1, 3].

A patient who desires to maintain and improve their physical, psychological and social well-being will tend to use CAM [4]. With the rising trend of dengue fever (DF) cases from 1999 to 2007 [5] and high incidence rates of dengue in Malaysia in 2011 [6], more patients will look up to CAM as an alternative form of treatment. Furthermore, a fool proof antiviral treatment for DF currently does not exist [7] and the development of safe tetravalent dengue vaccine is still undergoing clinical trials [8–10]. Therefore, the treatment is only supportive and symptomatic in nature. In addition, DF also carries the risk of developing into dengue haemorrhagic fever where the mortality rate is up to 44 % [11]. Living with these circumstances, patients began to realise that they need to take action to manage their condition by taking CAM [4].

For decades, several studies have shown the positive effect of CAM for DF treatment. Especially the fresh papaya leaf extracts [12, 13], bitter melon (*Momordica charantia*), green chirayta (*Andrographis paniculata*) [14], boneset (*Eupatorium perfoliatum*) [15]. Double-boiled frog with bitter gourd soup and the whole crab soup are the famous dishes used as CAM for treating DF among Chinese ethnics in Malaysia [16].

Although the scientists and the various governments are developing the effective prevention and control measures, including antiviral drugs and vaccines, CAM could provide good antiviral results as compared to their synthetic analogues [17, 18]. There is a necessity to rethink over medical education, increase the understanding of the concept of medicine and integrating CAM therapies in mainstream health care services [19]. However, the usage of CAM for DF treatment through safe and effective technologies are strongly recommended. Thus, this study aims to determine the prevalence, types, reasons, expenditure, and resources of CAM use among patient with DF.

## Methods

### Setting

This is a cross-sectional study of patient with DF attending the dengue clinic at Hospital Serdang, Hospital Kajang and Hospital Kuala Lumpur, Selangor, Malaysia. The sample was collected one off.

### Inclusion criteria

All patients above 18 years of age presented with a history of fever and evidence of thrombocytopenia (platelet count <150 × 10^9^/L) with or without dengue seropositivity were eligible for the study. To the best of our knowledge, there was no previous study on the prevalence of CAM use among patients with dengue. The sample size was calculated using Epi Info 7.0. The pilot study were conducted among 30 patients using the questionnaire to test for the patient’s understanding and to anticipate any potential problem particularly in term of response rate, from there we also managed to calculate the sample size. The sample were excluded from the analysis later on.

Based on the results of our pilot study, the prevalence of CAM use was 53.3 %. The estimated sample size was 269 with 90 % power, 95 % confidence interval (CI) and statistical significance level (α) at 5 %. The total number of respondents required was 309 after considering a non-response rate of 15 %. Universal sampling method was used to recruit the patients in the dengue clinic at Hospital Serdang, Hospital Kajang, and Hospital Kuala Lumpur.

### Data collection

A face-to-face interview was conducted by using a questionnaire. A written consent form was obtained from the respondents. The questionnaire was designed to capture the information on patients’ socio-demographic data, co-morbidity, types of CAM use, reasons for CAM use, resources consulted and the amount of expenditure on CAM (Refer to Additional file 1 on questionnaire). The survey was carried out during the first 3 weeks of July 2014.

### Practical definition

A dengue patient was defined as someone who had the history of fever and clinically diagnosed with thrombocytopenia with platelet count <150 × 109/L with or without dengue seropositivity. Dengue seropositivity was not used as a must criteria to diagnose dengue in this study because the serologically test only confirmed cases of dengue by approximately 40–50 % at the time of notification and fever accounts for almost 95 % of all reported cases [5].

In this study, CAM use was defined as the consumption of at least one out of the five types of CAM therapies; biological-based therapies (herbal and dietary supplements), alternative medical systems (including acupuncture, Ayurvedic and homeopathy), energy therapies (such as Qi Gong or Reiki), manipulative and body-based therapies (such as chiropractic care and massage) and mind (body intervention therapies (such as shamanism and yoga) [1, 3].

### Data analysis

Statistical analysis was conducted by using the IBM statistical package for the social sciences (SPSS) version 21.0. Categorical data were reported as proportions (percentage). Chi-square test or Fisher exact test was used for the categorical or dichotomous variables. Multiple logistic regression analysis was used to look for predictors of CAM use among patient with DF. All variables with the *p* value less than 0.25 in the simple logistic regression as well as clinically significant variables were entered into the multiple logistic regression. The dependent variable was CAM use. The independent variables were age, education level, duration of DF and household income. After adjustments were made for possible confounding factors (model 1: religion, ethnicity, education level and total family income) model 2: religion, ethnicity, education level, total family income and duration of dengue fever), multiple logistic regression were performed. We hypothesized that patient with low education level are tend to use CAM compared to those with higher education level. All analyses were made with 95 % CI, and the level of significance was set at *p* < 0.05.

### Ethical approval

Ethical approval was obtained from the Ethics Committee of National Malaysia Research Registry (NMRR-14-420-20622).

## Results

A total of 326 patient with DF were eligible for this study. Out of these, 20 patients refused to participate in the study. In the end, 306 patients with DF were enrolled into the study with the response rate of 93.9 %. Table 1 compares the characteristics of those with and without CAM use. Overall, the median age of the patients was 28 years with 58.4 % men. Most of them came from the white collar category with a background of secondary education (51.6 %) and monthly family income of RM 3000 (USD 803). Most of the respondents were categorized into classic DF (98.7 %) and a minority of them had dengue hemorrhagic fever (1.3 %). The mean duration of DF was 6 days. During univariate analysis, we found that the level of education (*p* = 0.01), monthly family income (*p* = 0.03) and the duration of DF had an association with CAM use (*p* = 0.02).

**Table 1:** Comparison in clinical variables between suspected dengue patient with or without CAM use (*N* = 306)

Socio-demographic factors	CAM Users *n* = 261, (%)		Non-CAM Users *n* = 45, (%)		*p*-value
Male	156	(86.7)	24	(13.3)	0.42
Female	105	(83.3)	21	(16.7)	
Ethnicity					
Malay	209	(86.7)	32	(13.3)	0.17
Non Malay	50	(80.0)	13	(20.0)	
Religion					
Muslim	210	(86.8)	32	(13.2)	0.15
Non-Muslims	51 (79.7)	(64.7)	13 (0.3)	(35.3)	
Level of Education					
Primary	7	(58.3)	5	(41.7)	0.01*
Secondary	133	(84.2)	25	(15.8)	
Tertiary	121	(89.0)	15	(11.0)	
Occupation					
No Occupation	75	(77.6)	16	(17.6)	0.37
Blue Collar	67	(85.9)	11	(14.1)	
Non-blue Collar	119	(86.9)	18	(13.1)	
Age (years)					
18–27	119	(85.0)	21	(15.0)	0.87
28–37	85	(86.7)	13	(13.3)	
> 38	57	(83.8)	11	(16.2)	
Monthly Family Income (RM)					
0–2500	113	(81.3)	26	(18.7)	0.13
2501–5000	109	(87.2)	16	(12.8)	
> 5001	39	(92.9)	3	(12.0)	
Duration of dengue fever (days)					
1–3	18	(72.0)	7	(28.0)	0.13
4–6	161	(85.6)	27	(14.4)	
> 7	82	(88.2)	11	(11.8)	

*There is significant association if *p*-value < 0.05

### Types of CAM used by patient with DF

Total CAM use of the patient with DF was 85.3 % (*N* = 261) with 95 % CI 0.81–0.89. Table 2 shows the type of CAM used by patients with DF. Biological therapies that involved dietary supplements (98.1 %) and herbal products (22.2 %) were the most commonly used. The most popular type of CAM was isotonic drink (85.8 %), followed by crab soup (46.7 %), papaya leaf extract (22.2 %), coconut juice (20.3 %) and watermelon (17.2 %).

**Table 2:** Type of CAM used by patients with dengue fever (*N* = 261)

Type of CAM	Frequency *n* = 261	Percentage (%)
Biological-based therapies	261	100.0
Herbal Supplement	58	22.2
Papaya leaf extract	58	22.2
Dietary Supplement	256	98.1
Isotonic drink	224	85.8
Crab soup	122	46.7
Coconut Juice	53	20.3
Watermelon	45	17.2
Chicken soup	25	9.6
Vitamin Supplement	21	8.0
Guava Juice	11	4.2
Boiled frog meat with bitter gourd	8	3.1
Other Juices	26	10.1
Alternative medical system	1	0.4
Energy therapy	0	0.0
Manipulative and body-based systems	0	0.0
Mind-body intervention	12	4.6

### Attitudes, beliefs and perceptions towards CAM

The mean duration of CAM use was 4 days. Table 3 shows the reasons, resources and expenditure of using CAM among patient with DF. One-third of the patients consumed CAM because they had a good impression from other CAM users. Another one-third of them just wanted to try it (32.2 %). About one-fifth (22.6 %) of them took it under the influence of family members. Most of the patients received information on CAM from their families (54.8 %), followed by friends (31 %), doctors (9.6 %), neighbors (2.3 %) and media (2.3 %). During DF, the median expenditure on CAM was RM 16.50 (USD 4.42) and more than half (57.1 %) of the patients spent less than RM 20.00 on CAM.

**Table 3:** Reasons for CAM use, source of information and expenditure spend on CAM by patients with dengue fever (*N* = 261)

Variables	Frequency (*N*)	Percentage (%)
Reason for using CAM		
• Have good impression on CAM from other CAM users	87	33.3
• Just want to try	84	32.2
• Due to family belief and tradition	59	22.6
• Lesser side effects than conventional medicines	9	3.4
• Dissatisfied with conventional medicines	0	0
• Others	22	8.4
CAM Information Source		
• Family	143	54.8
• Friends	81	31
• Doctor	25	9.6
• Neighbour	6	2.3
• Media	6	2.3
Amount of expenditure (RM)		
• RM0.00-19.99	149	57.1
• RM20.00-39.99	65	24.9
• RM40.00-59.99	34	13
• > RM60.00	13	5

### Multiple logistic regression

Table 4 shows the results of multiple logistic regressions. In multiple logistic regression analysis, after adjusting for all the variables in the model, education was the main predictor. Dengue fever patients with a tertiary level are more likely to use CAM 5.8 (95 % confidence interval (CI 1.62–20.45) and 3.8 (95 % CI 1.12–12.93) times than secondary level and primary and below respectively.

**Table 4:** Predictors of CAM use among patients with dengue fever (*N* = 306)

Variables	OR	95 % C.I.		Sig.
		Lower	Upper	
Education level				0.02
Tertiary level	5.76	1.62	20.45	0.01
Secondary level	3.80	1.12	12.93	0.03
Primary and below	1.00			
Religion Muslim	1.16	0.05	24.75	0.92
Non-Muslims	1.00			
Category income				0.42
> RM5000	2.30	0.62	8.45	0.21
RM2501–RM5000	1.30	0.64	2.64	0.47
RM0–RM2500	1.00			0.33
Ethnicity Malay	1.62	0.78	3.37	0.19
Non-Malays	1.00			
Category duration				0.19
≥ 7 days	2.73	0.91	8.20	0.07
4–6 days	2.17	0.81	5.81	0.12
1–3 days	1.00			

## Discussion

Dengue is a severe threat in various countries and there is still no specific treatment. Researchers are keen to identify a potential antiviral drug, generic compounds and natural products against dengue infection [7, 18]. On the other hand, people have started using CAM such as the extracts of *Carica papaya* and other dietary complements, as they believe that CAM is more effective against dengue. Taking this into account, this study had been conducted on 261 CAM users and 45 non-CAM users among patient with DF. The prevalence of CAM use (85.3 %) among dengue patients was high in comparison with non-CAM user. This finding was consistent with the studies on CAM use against chronic diseases such as diabetes mellitus [20, 21] and hypertension [22]. It was also supported by another study on CAM use against acute diseases like asthma [23].

A study was conducted on the use of *Carica papaya* leaf juice in dengue patients and the results showed a significant increase in the platelet count of these patients [13]. Surprisingly, beverage like isotonic drinks (85.8 %) and food like crab soup (46.7 %) become the commonest CAM use in our study. Some of the patients claiming that having isotonic drink was useful, however, it shows no proof in reducing DF [24]. Despite it did not have any therapeutic effect in curing DF but it gives a supportive effect that helps the dengue patient to regain energy [25]. Also, people from various demographics believed that isotonic drink drinks were able to treat DF, although they did not know the mechanism of action [16]. However, this isotonic drink might also endanger the life of a diabetic patient if they over consume it during DF [24, 26].

Although many studies found that papaya leaf have a significant effect on DF [12, 13], only one fifth (22.2 %) of dengue patients in this study consumed it. It seems possible that this result is due to the bitter taste of the papaya leaf [27–29] which make it less favorable choice as patient with DF also suffered from poor appetite [30–33].

In this study, we found that one-third of the patient with DF used CAM because they had a good impression of CAM from other CAM users. This has usually been under the influence of other patients in the same hospital and they believed that CAM can relieve their symptoms and help them recover faster from DF. Another one-third of them just wanted to try it (32.2 %) and this was consistent with another local study on the reasons of CAM use in patients with chronic diseases [22]. This belief differed from another previous local study where the main reason for CAM use among patients with diabetes was the quality and safety of CAM [34]. In addition, we found that none of the CAM users were dissatisfied with the conventional medicines. This finding was consistent with another local study [20].

The main source of information on CAM use among patient with DF was family (54.8 %). These results differed from some published studies on CAM where most of the respondents obtained information on CAM principally from their friends [19]. During DF, the median expenditure on CAM was RM 16.50 (USD4.42) and more than half (57.1 %) of the patients spent less than RM 20.00 on CAM. This finding was in agreement with the local studies that showed that the expenditure on CAM was generally low [20, 22]. This finding further supported the previous published study where patients who tried to use CAM had lower monthly expenditures compared to those who did not use CAM [35].

In our study, those who obtained a tertiary level of education (89 %) were more likely to use CAM compared to others. This was due to their environment where educated people were more aware about the complications of low platelet count, which in turn caused them to look for a supplement including CAM. This result was consistent with another study, which demonstrated that individuals with higher educational levels had a better knowledge and perception towards certain diseases and this lead them to be more self-empowered in terms of their own health [32, 33].

### Strengths and limitations

The study would have been better if simple random sampling was used instead of universal sampling for all the respondents attended to dengue clinic. Ideally, data should be collected repeatedly during the illness and when the patients are fully recovered from DF to determine all the types of CAM use during the duration of a patient’s illness. However, it was done because of the lack of manpower and time constraint. Besides that the findings on association both univariate and multiple logistic regression have to be interpreted with cautious due to small number of sample. Thus future population based study with a bigger sample size is recommended to be carried out.

## Conclusion

The study demonstrated the CAM use among patients DF was high. A higher level of education was known to be the factor associated with CAM use. The most common CAM used among DF were isotonic drinks, crab soup and papaya leaf extract. More number of studies should be conducted in the future to identify the active compound in crab soup, which has an antiviral activity against DF.

## Additional file

Additional file 1:
**Questionnaire.** (DOC 44 kb)

## Abbreviations

CAM: complementary and alternative medicine.
DF: dengue fever
CI: confidence interval
