# Supplementary material for: Complementary alternative medicine use among patients with dengue fever in the hospital setting: a cross-sectional study in Malaysia
Source: BMC Complement Altern Med. 2016 Jan 29;16:37. doi: 10.1186/s12906-016-1017-0 (PMC4731995; doi:10.1186/s12906-016-1017-0)
Supplement: Additional file 1: — Questionnaire. (DOC 44 kb) [file 12906_2016_1017_MOESM1_ESM.doc]

Additional file 1

QUESTIONNAIRE/BORANG SOAL SELIDIK

SECTION A: SOCIODEMOGRAPHIC INFORMATION/

*BAHAGIAN A : INFORMASI SOSIODEMOGRAFI*

1. Gender/*Jantina*: _0_ Male/*Lelaki* _1_ Female/*Wanita*

2. Age/*Umur*: ______ years/*tahun*

3. Ethinicity/*Kaum*:

0 Malay/*Melayu* _1_ Chinese/*Cina* _2_ Indian/*India* _3_ Others/*Lain-lain*

4. Religion/*Agama*:

0 Muslim/*Islam* _1_ Buddhist/*Buddha* _2_ Hindu/*Hindu*

3 Christian/*Kristian* _4_ Sikh/*Sikh* _5_ Others/*Lain-lain*

5. Level of Education/*Taraf pendidikan*:

0 No education/*Tiada pendidikan* _1_ Primary/*Rendah*

2 Secondary/*Menengah* _3_ Tertiary/*PengajianTinggi*

6. Occupation/*Pekerjaan*: _________________________________

0 No occupation/*Tiada pekerjaan*

1 Blue collar/*Kolar biru* (relating to manual work or workers, particularly in industry/ berkenaan kerja manual atau kerja industri)

2 Non-blue collar/*Bukan kolar biru*

7. Family household income/*Pendapatan isi keluarga*: _____________________________

(Example/*Contoh:* Total income of husband, wife and children/ *Jumlah pendapatan suami, isteri dan anak sebulan*)

8. Type of dengue fever/*Jenis demam denggi*:

0 Classical Dengue Fever/*Demam Denggi Klasikal*

1 Dengue Haemorrhagic Fever/*Demam Denggi Berdarah*

9. Duration of disease/*Tempoh masa penyakit*: _____________days/*hari*

SECTION B: USAGE OF COMPLEMENTARY ALTERNATIVE MEDICINE (CAM)

*BAHAGIAN B: PENGGUNAAN PERUBATAN KOMPLEMENTARI DAN ALTERNATIF (PKA)*

1. Do you have any experience of CAM usage? (include supportive drink like 100 Plus)

*Adakah kamu mempunyai pengalaman dengan penggunaan PKA? (termasuk minuman 100 Plus)*

0 No/Tidak _1_ Yes/Ya

2. How long have you been the user of CAM? *Berapa lamakah kamu telah menjadi pengguna PKA?*: ____________days/*hari*

3. Which type of CAM had been you use? (can tick more than once)

*Apakah jenis PKA yang kamu telah gunakan? (boleh tanda lebih dari satu)*

| a) Papaya leaves extract/ *Ekstrak daun betik* |  | o) Acupuncture/*Akupuntur* |  |
| --- | --- | --- | --- |
| b) Crab soup/*Sup ketam* |  | p) Giloy or Amrita |  |
| c) Boiled frog meat with bitter gourd/*Katak rebus dengan peria katak* |  | q) Kakamachi syrup/*Sirap Kakamachi* |  |
| d) Coconut juice/*Air kelapa* |  | r) Homeotherapy/Homeopati - *Eupatorium perfoliatum* |  |
| e) Watermelon/*Tembikai* |  | s) Shamanism/*Bomoh* |  |
| f) Neem leaves/*Daun Neem* |  | t) Qi Gong/Reiki |  |
| g) Tawa-tawa tea/*Teh Tawa-tawa* |  | u) Religion/Agama |  |
| h) Sweet potato leaves/ *Daun ubi keledek* |  | v) Yoga |  |
| i) *Echinacea* |  | w) Music therapy/Terapi muzik |  |
| j) *Astragalus* *oldenseal* |  | x)Aromatherapy/Terapi aroma |  |
| k) Porcupine bezoar stone/*Batu bezoar landak* |  |  |  |
| l) 100 Plus drink/*Minuman 100 Plus* |  |  |  |
| m) Chicken soup/*Sup ayam* |  |  |  |
| n) Supplement (Vitamin C, Zinc,  Omega 3)/ *Pemakanan tambahan (Vitamin C, Zink, Omega 3)* |  |  |  |

Others with description:

*Lain-lain dengan penerangan:*

_________________________________________________________________ State the reasons why you use CAM. (Tick one only)

*Nyatakan sebab-sebab mengapa kamu mengunakan PKA. (Tanda satu sahaja)*

0 Dissatisfied with conventional medicines/*Tidak berpuas hati dengan perubatan konvensional (moden)*

1 Lesser side effects than conventional medicines/*Kurang kesan sampingan berbanding dengan perubatan konvensional*

2 Have good impression on CAM from other CAM users/*Mempunyai anggapan baik terhadap PKA kerana pengguna PKA lain*

3 Due to family belief and tradition/*Disebabkan kepercayaan dan tradisi keluarga*

4 Just want to try/*Hanya nak mencuba*

5 Others/*Lain-lain*: ________________________________________

6. What is your main source of information on (CAM) practice? (Tick one only)

Apakah sumber maklumat utama anda untuk PKA? (Tanda satu sahaja)

0 Doctor/*Doktor* _1_ Friends/*Kawan* _2_ Family/*Keluarga*

3 Neighbour/*Jiran* _4_ Media/*Media*

5 Others/*Lain-lain* (Specify/*Nyatakan*): ________________________________________________

7. State the average of expenditure that you spend for CAM (RM)?

*Nyatakan purata perbelanjaan yang kamu belanjakan untuk PKA set(RM)?*

___________________________________________________________________
